# Transcriptional regulation of the ABCC6 gene and the background of impaired function of missense disease-causing mutations

**DOI:** 10.3389/fgene.2013.00027

**Published:** 2013-03-11

**Authors:** Tamás Arányi, Caroline Bacquet, Hugues de Boussac, Marcin Ratajewski, Viola Pomozi, Krisztina Fülöp, Christopher N. Brampton, Lukasz Pulaski, Olivier Le Saux, András Váradi

**Affiliations:** ^1^Institute of Enzymology, Research Center for Natural Sciences, Hungarian Academy of SciencesBudapest, Hungary; ^2^Laboratory of Transcriptional Regulation, Institute of Medical Biology, Polish Academy of SciencesLodz, Poland; ^3^Department of Cell and Molecular Biology, John A. Burns School of Medicine, University of Hawai’i, HonoluluHI, USA

**Keywords:** ABCC6, HNF4α, 4-phenyl-butyrate, C/EBPβ, calcification, pseudoxanthoma elasticum, generalized arterial calcification in infancy

## Abstract

The human ATP-binding cassette family C member 6 (*ABCC6*) gene encodes an ABC transporter protein expressed primarily in the liver and to a lesser extent in the kidneys and the intestines. We review here the mechanisms of this restricted tissue-specific expression and the role of hepatocyte nuclear factor 4α which is responsible for the expression pattern. Detailed analyses uncovered further regulators of the expression of the gene pointing to an intronic primate-specific regulator region, an activator of the expression of the gene by binding CCAAT/enhancer-binding protein beta, which interacts with other proteins acting in the proximal promoter. This regulatory network is affected by various environmental stimuli including oxidative stress and the extracellular signal-regulated protein kinases 1 and 2 pathway. We also review here the structural and functional consequences of disease-causing missense mutations of ABCC6. A significant clustering of the missense disease-causing mutations was found at the domain–domain interfaces. This clustering means that the domain contacts are much less permissive to amino acid replacements than the rest of the protein. We summarize the experimental methods resulting in the identification of mutants with preserved transport activity but failure in intracellular targeting. These mutants are candidates for functional rescue by chemical chaperons. The results of such research can provide the basis of future allele-specific therapy of ABCC6-mediated disorders like pseudoxanthoma elasticum or the generalized arterial calcification in infancy.

## THE ABCC6 PROTEIN

ATP-binding cassette family C member 6 (ABCC6) encodes an ATP-dependent transporter primarily found in the plasma membrane of hepatocytes (Madon et al., 2000). *In vitro* studies supports that it functions as an organic anion efflux pump (Belinsky et al., 2002; Iliás et al., 2002) transporting (an) unidentified substrate(s) from the liver toward the circulation. The functional relationship between ABCC6 and ectopic calcification is not understood.

**FIGURE 1:**
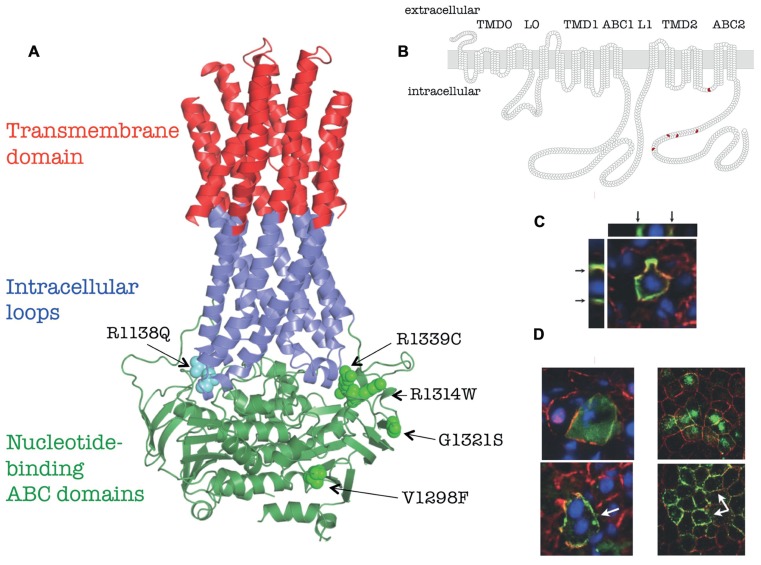
**Membrane topology, homology model and *in vivo* localization of human ABCC6 and its mutants (based on Le Saux et al., 2011)**. **(A)** Homology model of human ABCC6. The position of the missense mutants studied are indicated. **(B)** Membrane homology model of human ABCC6. The domain structure are indicated on the top, the position of missense mutants are shown in red. **(C)** Colocalization of the endogenous mouse Abcc6 (red) and human ABCC6 (green) *in vivo* in mouse liver after HTVI-delivery of ABCC6 cDNA by confocal microscopy. Arrows indicate the basolateral localization on Z-stack cross/sections. **(D)** Effect of 4-PBA on membrane targeting of Q1314W. *Left panel*: colocalization of the endogenous mouse Abcc6 (red) and human ABCC6 mutant (green) *in vivo* in mouse liver after HTVI-delivery of ABCC6 cDNA without 4-PBA treatment (*upper panel*) or with 4-PBA treatment (*lower panel*). *Right panel*: colocalization of the endogenous dog Na/K-ATPase (red) and human ABCC6 mutant Q1314W (green) *in vitro* in polarized MDCKII cells without 4-PBA treatment (*upper panel*) or with 4-PBA treatment (*lower panel*). White arrows point to the altered membrane localization.

On the basis of sequence similarity forty-eight ABC proteins are annotated in the human genome and classified as members of seven subfamilies. The ABC proteins share the general structural features: they harbor two nucleotide-binding (ABC) domains and two transmembrane domains (TMDs), each with six membrane-spanning helices (the so called “core structure”). The ABCC-subfamily consists of 12 proteins; most of them are active transporters. Two additional domains are fused to this core structure N-terminally in some ABCC-type proteins (“long MRPs” like ABCC1, 2, 3, 6, 8, 9, and 10): a TMD with five membrane embedded helices and an intracellular approximately 80 amino acid long loop. The domain architecture of this type of proteins, including ABCC6, can be described like TMD0-L0-TMD1-ABC1-L1-TMD2-ABC2 [L0 and L1 are intracellular loop (ICL); **Figure 1B**]. ABCC6 consists of 1503 amino acids and *in vitro* studies demonstrated the transport of different organic anions, glutathione-conjugates like glutathione *S*-conjugated leukotriene C4 (LTC4), *N*-ethylmaleimide *S*-glutathione (NEM-GS), and *S*-(2,4-dinitrophenyl) glutathione (Iliás et al., 2002), while the rat ortholog transports an anionic cyclopentapeptide (Belinsky et al., 2002). It was found using an *in vitro* transport assays that three of the missense mutations described as causative variants in pseudoxanthoma elasticum (PXE) result in the loss of ATP-dependent transport of test substrates (Iliás et al., 2002). It has also been suggested that overexpression of ABCC6 is able to confer low levels of resistance to several commonly used natural product anticancer agents like etoposide, doxorubicin, daunorubicin, and actinomycin D (Belinsky et al., 2002). However, clinically relevant ABCC6-mediated drug resistance has never been found.

Since the first PXE-causing mutations were discovered (Bergen et al., 2000; Le Saux et al., 2000, 2001; Ringpfeil et al., 2000), the number of identified disease-causing variants has exceeded 350. By searching PubMed papers reporting ABCC6 mutations and polymorphisms an internet-based mutation/variation database of ABCC6 has been established. Upon creating the database approximately 300 published mutations in 51 publications by 23 corresponding authors were collected and 65 potential errors in the reported mutations or sequences were found. Only corrected (confirmed) mutations are included into the database (Váradi et al., 2011), which contains now nearly 400 mutation/variations of the gene (http://www.ncbi.nlm.nih.gov/lovd/home.php?select_db=ABCC6). The majority of the mutations are missense. Despite the large number of PXE-specific mutations that has been identified in ABCC6, no clear genotype–phenotype correlation has emerged.

No high-resolution three-dimensional structure of ABCC-type is available. However, a three-dimensional homology model of ABCC6 is already built and published (Fülöp et al., 2009), made possible by using the coordinates of high-resolution crystalline structures of other ABC proteins (Dawson and Locher, 2006; Aller et al., 2009). Newly recognized structural elements are the long “rigid” extensions of the transmembrane helices, called ICLs were also obvious in the homology models. Each half of the ABC proteins has two ICLs interacting with the ABC domains. The coupling helices contact with their “own” as well as with the “opposite” ABC domains, hence a special type of domain swapping can be recognized in the structure (see insert on **Figure 1A**). The homology models constructed can be interactively studied using the ABCC6 database.

The disease-causing mutations in the protein are distributed on an uneven fashion and performing statistical analysis significant clustering of the missense PXE mutations was found at the domain–domain interfaces: at the transmission interface that involves four ICLs and the two ABC domains as well as at the ABC–ABC contact surfaces. In the nucleotide-saturated model the mutations affecting these regions are 2.75- and 3.53-fold more frequent than the average mutational rate along the protein sequence, respectively (Fülöp et al., 2009). At the predicted ICL–ABC interfaces in the nucleotide-free model the mutational rate is 4.25-fold more frequent than the average mutational rate along the protein sequence (the ABC domains are distant in this conformation; Váradi et al., 2011). The observed significant clustering means that the domain contacts are much less permissive to amino acid replacements than the rest of the protein. These results provide a “bridge” between genetic data and protein structure and can be viewed as novel proof of the importance of the studied domain–domain interactions in the ABCC6 transporter.

A systematic experimental study has been initiated to investigate the impact of missense disease-causing mutation in order to better understand the molecular failure triggered by different mutations. Amino acid substitutions in large plasma membrane proteins such as ABCC6 generally result in decreased activity, major conformation changes, low level of plasma membrane targeting or a combination thereof. Therefore, studying the consequences of naturally occurring disease-causing missense mutations can provide important insights into the relationship between protein structure and function, which may later assist in the development of therapeutic applications (Le Saux et al., 2011). We review here the major steps of this systematic experimental study.

The complex experimental repertoire consists of expression and biochemical characterization of the protein variants, investigation of their plasma membrane vs. intracellular localization in polarized cell cultures. Finally, investigation the *in vivo* stability and cellular location of mutated ABCC6 in fully differentiated hepatocytes by transiently expressing the human mutant proteins was performed in the liver of C57BL/6J mice. Five missense mutations, V1298F and G1321S in the C-proximal ABC domain and R1138Q, R1314W, and R1339C at the transmission interface were included into the study. The location of the mutated residues is illustrated on **Figure 1A**. An N-terminal truncated mutant, del_1__-__277_ABCC6 (DABCC6) missing the TMD_0_ and L_0_ domains has also been constructed as a control (see **Figure 1B**). The results are summarized in **Table 1**. In detail: the wt ABCC6 is fully functional in the biochemical transport and enzymatic (MgATP-binding and catalytic intermedier formation) assays, it is integrated into the basolateral membrane of polarized Madin–Darby canine kidney type II (MDCKII) cells *in vitro* as well as that of the mouse hepatocytes *in vivo*. The DABCC6 appears to be an ideal negative control as it is inactive, and localized intracellularly both in MDCKII cells and in mouse liver cells.

**Table 1:** Function and intracellular localization of ABCC6 variants.

ABCC6 variant	Stability in Sf9	MgATP-binding	ATPase catalytic intermediate	Transport activity (% of WT)	Plasma membrane localization in MDCKII cells	Plasma membrane localization in mouse liver	Intracellular localization in mouse liver
WT	Stable	Yes	Yes	100	+++++	+++++	-
DABCC6	Stable	n.d.	n.d.	<10	-	-	+++++
R1138Q	Stable	Yes	Yes	~85	++++	++	+++
V1298F	Stable	Yes	No	<10	+++++	+++++	-
G1321S	Stable	Yes	No	<10	-	-	+++++
R1314W	Stable	Yes	Yes	~90	-	+	++++ (ER)
R1339C	Unstable	n.a.	n.a.	n.a.	-	-	+++++

*n.d., not determined; n.a., not applicable; ER, mostly retained with the endoplasmic reticulum.*

*The symbol “+” represents semiquantitative estimate of the presence of immunohistochemical signal in the given compartment (+++++ is maximal abundance).*

*The symbol “-” represents absence of immunohistochemical signal in the given compartment.*

The R1139C mutant did not result in native size protein when expressed in Sf9 cells indicating folding problems. Two of the variants (V1298F and G1321S) were found to be inactive, though they were capable of binding MgATP, their capacity to form a catalytic intermediate (“occluded nucleotide state”) was impaired. R1138Q and R1314W were fully active in the transport assay. When overexpressed in MDCKII cells surprisingly, R1139C show a level of protein expression similar to the wild type, however, it was found intracellularly, indicating that the mutation triggered an altered conformation not compatible with plasma membrane targeting. The same was true for mutants G1321S, R1338Q, and R1314W, while the inactive V1298F was found mostly in the plasma membrane.

ATP-binding cassette transporters are traditionally studied in cultures of kidney-derived MDCKII cells, but these are not ideal for studying hepatic proteins, as they do not correspond to the physiology of the liver where ABCC6 is primarily present. To overcome these obstacles hydrodynamic tail vein injection (HTVI) method was utilized to study the human ABCC6 protein in the fully differentiated liver of a living mouse. This method delivered DNA to the liver very effectively (Liu et al., 1999; Zhang et al., 2004) and ensured the selective hepatic expression of the wt human protein in mouse liver with an adequate basolateral targeting (see **Figure 1C**).

The *in vivo* intracellular localization of the human variants was also studied. Only the inactive V1298F mutant showed plasma membrane localization at a similar high level as the wt. The R1339C and the G1321S proteins were found intracellularly (similar to the results obtained in the *in vitro* cell culture system). R1138Q and R1314W showed mostly intracellular appearance, however, a portion of the protein could reach the plasma membrane.

From the above the functional features and the pathogenic character of the mutants can be delineated. They clarify the biochemical and cellular effects of ABCC6 mutations that lead to dystrophic calcification in humans. Most probably the dramatically reduced transport activity of mutants V1298F and G1321S is the molecular background of the disease in patients with these variants (irrespective that in the case of the former one the correct plasma membrane targeting is preserved). In the case of R1338, R1314, and R1339 the aberrant folding resulted by the mutation prevents plasma membrane localization, which seems to be the prerequisite of the normal physiological function (irrespective that two of these mutants are active as organic anion transporter).

The successful completion of these experiments may provide valuable basic results and those in the field of translational medical research. They indicate the potential for therapeutic interventions to correct the defects of a mutant ABCC6 protein. Mutants with preserved transport activity but failure in intracellular targeting are candidates for functional rescue.

Several studies have shown that sodium 4-phenylbutyrate (4-PBA) can act as a chemical chaperone (e.g., Rubenstein and Zeitlin, 2000; van der Velden et al., 2010) for misfolded proteins in the endoplasmic reticulum (ER). Therefore, it was studied whether pre-treating mice with 4-PBA before HTVI would restore normal cellular trafficking of those mutants that retained transport activity. R1138Q and R1314W were tested, along with R1339C and the WT protein as non-functional and functional controls respectively. It is expected that those mutant proteins retained in the ER are to be rescued by 4-PBA. We have found that R1314 shows ER retain, as it colocalized with an ER-marker in mouse hepatocytes, while R1338Q and R1339C was not found in this location.

Oral treatment of the animals with 4-PBA before HTVI resulted in no effect on the intracellular localization of R1338Q and R1339C. However, treatment of mice with 4-PBA improved the cellular localization of R1314W (**Figure 1D**, left panels), which was confirmed in MDCKII cells (**Figure 1D**, right panels). This result suggests that the incorrect intracellular trafficking of this otherwise functional mutant is due to ER retention, implicating protein misfolding, and that correct intracellular trafficking of an otherwise functional mutant can be restored. As 4-PBA is an FDA-approved drug, these results may facilitate further clinical research for allele-specific therapy of PXE.

## TRANSCRIPTIONAL REGULATION OF THE ABCC6 GENE

However, we should also keep in mind that PXE is not a single disease but a group of similar phenotypes, which is constituted by PXE, PXE-like diseases and generalized arterial calcification of infancy (GACI; Aessopos et al., 1992, 2008; Vanakker et al., 2007; Nitschke et al., 2012) based on various, mostly unknown pathomechanisms. Therefore, another therapeutical approach for at least some PXE(-like) diseases could be targeted to the gene instead of the misfolded protein. In order to explore this idea we have to go back to the transcriptional regulation of *ABCC6*.

Although some studies have reported ubiquitous expression of ABCC6 (Beck et al., 2003, 2005), there is a consensus in the literature that the gene is primarily expressed in the liver and to a much lower extent in the kidneys (Kool et al., 1999; Madon et al., 2000; Beck et al., 2005; Maher et al., 2005, 2006). There is also intestinal expression of the gene based on the data published by (Kool et al., 1999) and findings in human cell lines (Ratajewski et al., 2012). This expression pattern is similar in rodents and human even if the gene is in a dynamically evolving genomic region (Eichler et al., 2007; Symmons et al., 2008). This fast evolution is characterized by numerous recent segmental duplications but also some primate-specific insertions/deletions with uncovered regulatory role, in spite of the relatively high conservation level of the coding region and some other regulatory sequences (Eichler et al., 2007; Symmons et al., 2008; Ratajewski et al., 2012). Why the gene is expressed essentially in the liver and to a lower level in very few other tissues, while in the organs suffering from the loss-of-function ABCC6 mutations ABCC6 is normally not expressed? To formulate this question in a different way: what is responsible for this intriguing tissue-specificity?

To answer this question a battery of molecular biological tools and assays were used, e.g., luciferase reporter gene assay, gel shift assay (EMSA), transcription factor microarray, DNase I hypersensitivity assay (DHA), and chromatin immunoprecipitation (ChIP). Bioinformatic studies revealed the existence of two evolutionarily well-conserved sequence blocks: one in a distal 5′ region, near to the neighboring NOMO3 gene, and one in the proximal promoter of the *ABCC6* gene (Arányi et al., 2005). Similar data were reported in human cell lines (Arányi et al., 2005) and mouse tissues (Douet et al., 2007) showing that in the proximal promoter, the presence of silencing epigenetic factors, namely DNA methylation, correlates inversely with the expression level of the gene, suggesting that the regulation of tissue-specific expression is determined by this region.

An important number of luciferase reporter gene assays were performed to find the regulatory elements in the promoter and later for the identification of transcription factors binding those elements (Arányi et al., 2005; Douet et al., 2006, 2007; Jiang et al., 2006; Ratajewski et al., 2006, 2008, 2009, 2012; de Boussac et al., 2010; Martin et al., 2011). In these experiments the firefly luciferase gene is under the transcriptional control of a target sequence in a plasmid vector. After transient transfection of the plasmid the transcriptional regulatory capacity of the target sequence is estimated by the luminometric luciferase enzymatic activity measurement. The use of this technique revealed a DNA methylation-dependent activatory (-332/+72 relative to the translation start site) and a repressor region (-718/-332; Arányi et al., 2005; Jiang et al., 2006). The -332/+72 fragment turned out to be composed of a -145/+72 core promoter (characterized by a ubiquitous low activity in the assay in all cell types tested and not sensitive to DNA methylation (Arányi et al., 2005; Ratajewski et al., 2006, 2012; de Boussac et al., 2010) and two other fragments located between -209/-145 and -233/-209 both sequences active in a cell type-specific manner (Ratajewski et al., 2009, 2012; de Boussac et al., 2010). Furthermore, it has also been shown that the activity of the primate-specific -233/-209 element depends on the activity of the -209/-145 sequence (Ratajewski et al., 2009, 2012).

Another set of luciferase reporter gene assays were carried out based on the results obtained by DHA. This classical method reveals accessible regions to partial DNase I digestion in intact nuclei (Lu and Richardson, 2004). The accessible regions frequently represent active transcriptional regulatory elements. Surprisingly, the data suggested that not only the proximal promoter but also the first intron of the human *ABCC6* gene might have an important tissue- or cell type-specific regulatory role in the control of the expression of the gene (Ratajewski et al., 2012). Later on, with the help of the luciferase assays the presence of an important primate-specific sequence in the first intron of the gene (+629/+688) was found, which multiplies by several fold the transcriptional activity of the proximal promoter alone (Ratajewski et al., 2012). This intronic sequence and the proteins binding to turned out to depend on the presence of the proximal promoter and more specifically on the -233/-209 element, with which it might interact directly. Interestingly both sequences are primate-specific. These two elements also interact with the protein binding the sequence -209/-145, which is an evolutionarily highly conserved fragment (Ratajewski et al., 2012).

In another series of experiments, the different proteins recognizing the response elements were identified. Either luciferase assays with mutated target regulatory sequences or luciferase plasmids co-expressed with candidate transcription factors were used (Douet et al., 2006; Jiang et al., 2006; Ratajewski et al., 2006, 2008, 2009, 2012; de Boussac et al., 2010). Another approach was the EMSA, when the *in vitro* binding of a protein from a nuclear extract or its recombinant form is tested. In these cases in general short, labeled oligonucleotides are used as probe and in case of binding a protein, upon acrylamide gel electrophoresis a delay in the migration can be observed relative to that of the free probe (Douet et al., 2006, 2007; Jiang et al., 2006; Ratajewski et al., 2008, 2009). The transcription factor array is based on a similar principle [the hybridization of DNA and specific transcription factors (Jiang et al., 2006; Martin et al., 2011). Finally, ChIP can immunoprecipitate a target protein reversibly linked to its target sequence in the natural chromatin context after fragmentation of the chromatin. The immunoprecipitated fraction can then be tested by qPCR to determine the binding of the target protein to the sequences of interest (Douet et al., 2006, 2007; Ratajewski et al., 2006, 2008, 2012; de Boussac et al., 2010; Martin et al., 2011)]. These techniques led different groups to demonstrate the crucial regulatory role of hepatocyte nuclear factor 4 (HNF4) both in the human (de Boussac et al., 2010) and mouse (Douet et al., 2006) *ABCC6* gene. HNF4 binds to an evolutionarily highly conserved degenerate site between -209/-145 (de Boussac et al., 2010; Ratajewski et al., 2012). These data demonstrate the role of this transcription factor, which is a master regulator of metabolic genes in the liver. Interestingly, ABCC6 is expressed only in tissues where HNF4 is also present, suggesting that this transcription factor determines the tissue-specific expression of ABCC6. Our data also showed that in spite of the very important role of HNF4, it does not confer very high transcriptional activity to the gene. Further experiments suggested the binding of several other transcription factors to the proximal promoter [PLAG family members (Ratajewski et al., 2008, 2009), RXR (Ratajewski et al., 2006), SP1 and TGF-β (Jiang et al., 2006), and SP1 and NF-E2 in mouse (Douet et al., 2006, 2007; Martin et al., 2011)], however, the potential role of these different proteins is still unclear. The important role of CCAAT/enhancer-binding protein beta (C/EBPβ) in activating the transcription of the gene by binding the primate-specific sequence in the first intron was also shown (Ratajewski et al., 2012). Our data also suggest the binding of C/EBPα (unpublished).

**FIGURE 2:**
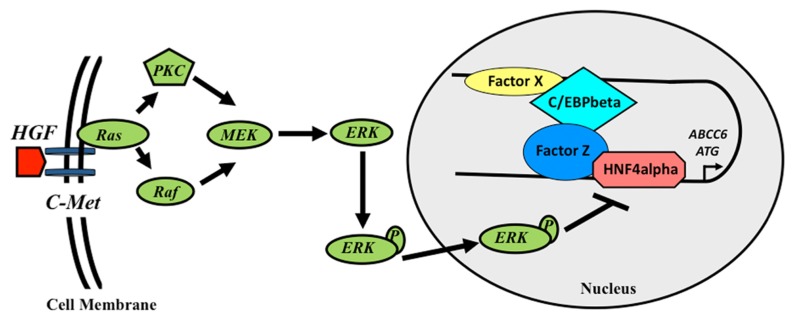
**Schematic model of the transcriptional regulation of the human ABCC6 gene**. Growth factors inhibit the expression of ABCC6 via the activation of ERK1/2 cascade, which reduces HNF4α binding to the promoter. In the intron, a transcriptional activator sequence binds C/EBPβ and another hypothetical protein (Factor X). These proteins interact with a complex formed by HNF4α and another factor (Y) binding the promoter, activating the transcription of the gene.

In the previous sections we have summarized the regulatory elements in the ABCC6 gene and the transcription factors binding them. Altogether this network governs the tissue-specific transcriptional regulation of the expression of the gene. However, there is a third layer of investigations, which focused on the regulation of the *ABCC6* gene expression by environmental factors. These experiments showed that the extracellular signal-regulated protein kinases 1 and 2 (ERK1/2) cascade negatively regulates the expression of the gene (de Boussac et al., 2010). It was shown that this inhibition converges on HNF4, which due to the activation of ERK1/2 looses its activator potential in two phases. Similarly, it was shown that oxidative stress inhibits the expression of the gene in human cell lines. This effect is probably also at least partially acting via the ERK pathway (de Boussac et al., 2010). The role of oxidative stress in the downregulation of the mouse *Abcc6* gene expression was shown in a beta-thalassemia model mouse. In this model the role of NF-E2 transcription factor was described (Martin et al., 2011). Finally, the oxidative stress factors or other agents present in the serum of PXE patients were shown to downregulate the expression of the human gene in cell culture systems. The detailed molecular mechanism behind this observation remains to be deciphered (Le Saux et al., 2006). Altogether these findings described in these sections above are summarized on (**Figure 2**).

What are the future questions in the field? Can we use the obtained information either in the better understanding or in the cure of the disease? The major, still remaining question in the PXE field is the identification of the molecule the ABCC6 protein is transporting. Although, the better understanding of the transcriptional regulation of the gene will not directly provide an answer to this question however, it might give some hints. For example, since the gene is downregulated by reactive oxygen species (ROS) it probably does not play a role in the transport of molecules participating in the ROS pathways. Similarly, as HNF4 and C/EBP transcription factors regulate the expression of metabolic genes, this might suggest that ABCC6 transports a common metabolite. Concerning the use of the knowledge obtained from these studies in the clinics, the situation is encouraging as in various clinical conditions [carriers of PXE mutations (Köblös et al., 2010; Vanakker et al., 2011) and secondary PXE (Aessopos et al., 1992, 2008)] the increase of the expression of the ABCC6 gene could lead to an improvement of the symptoms. Therefore, we consider that continuing the research to find the molecular mechanisms governing the regulation of the ABCC6 expression will lead to both a better understanding of the pathomechanism of the disease and an improvement of PXE related clinical phenotypes.

## Conflict of Interest Statement

The authors declare that the research was conducted in the absence of any commercial or financial relationships that could be construed as a potential conflict of interest.
